# The Impact of Residency and Urbanicity on *Haemophilus influenzae* Type b and Pneumococcal Immunization in Shanghai Children: A Retrospective Cohort Study

**DOI:** 10.1371/journal.pone.0097800

**Published:** 2014-05-14

**Authors:** Abram L. Wagner, Xiaodong Sun, JoLynn P. Montgomery, Zhuoying Huang, Matthew L. Boulton

**Affiliations:** 1 Department of Epidemiology, School of Public Health, University of Michigan, Ann Arbor, Michigan, United States of America; 2 Department of Immunization Program, Shanghai Municipal Center for Disease Control and Prevention, Shanghai, China; 3 Division of Infectious Diseases, School of Medicine, University of Michigan, Ann Arbor, Michigan, United States of America; Fudan University, China

## Abstract

**Background:**

*Haemophilus influenzae* type b (Hib) vaccine and pneumococcal conjugate vaccine (PCV) are relatively expensive, newly introduced vaccines in China. This study evaluates the impact of residency and urbanicity on Hib vaccine and PCV coverage for children aged 2 to 7 years living in Shanghai, China, in August 2012.

**Methods:**

In this exploratory cohort study, a sample of children aged 2 to 7 years, all of whom were eligible to have received the complete series of Hib vaccine and PCV, was obtained from the Shanghai Immunization Program Information System. Three measures of vaccination coverage for Hib vaccine and PCV were examined: dose 1 coverage, series completion, and timeliness of dose 1 vaccination. Multivariable binomial regression was used to estimate the difference in vaccination coverage between locals and the floating population.

**Results:**

Dose 1 coverage was 50.9% for Hib vaccine and 11.4% for PCV for the 28,141 abstracted pediatric records. For both vaccines, dose 1 coverage was higher in locals than in the floating population. The disparity in coverage between locals and the floating population was greater in suburban areas than urban areas. Of all children who received dose 1, 79.7% completed the Hib vaccine series, and 91.3% completed the PCV series. Timely dose 1 coverage was 8.2% for Hib vaccine and 0.5% for PCV.

**Conclusion:**

Low vaccination coverage and extremely low levels of timely dose 1 vaccination indicate that current vaccination efforts are inadequate to reduce the burden of Hib and pneumococcal disease among Chinese children, especially infants. Government funding of the Hib vaccine and PCV through the Expanded Program on Immunization would increase uptake and could also ensure that improvement in the timeliness of administration and series completion is targeted for all demographic groups.

## Introduction

The bacteria *Haemophilus influenzae* type b (Hib) and *Streptococcus pneumoniae* (pneumococcus) are important causes of pediatric illness [1]–[3] and are associated with invasive clinical disease comprising pneumonia, meningitis, and bacteremia [4]. Globally, an estimated 199,000 children under 5 years of age died from invasive Hib disease and 476,000 children under 5 years of age died from invasive pneumococcal disease in 2008 [4].

The epidemiology of childhood Hib and pneumococcal disease in China is not well characterized [5]. Chen et al. estimate over 261,000 cases of pneumococcal pneumonia and meningitis occur annually in China in children under 5 years of age and approximately 11,000 die from these diseases [6], while Watt et al. estimate 19,000 childhood deaths from Hib occur each year in China [7]. The burden of disease is higher in marginalized populations; pneumonia incidence is higher in rural areas than urban areas [8]. As China rapidly urbanizes, migrants from rural areas, the so-called “floating population,” relocate to wealthier cities to find work. Children in the floating population are at greater risk of morbidity and mortality from infectious diseases than local children [9]–[11]. The Chinese government has prioritized reducing this disparity in infectious disease risk through vaccination [12].

Highly effective vaccines can prevent both Hib- and pneumococcal-related disease when administered on time with a multi-dose series [13]. The Hib conjugate vaccine (Hib vaccine) was licensed in 1987 in the US [14] and has been available in China since 2000 [10]. The pneumococcal conjugate vaccine (PCV) has been available starting in 2000 in the US [15] and since 2008 in China [16]. The PCV formulation currently available in China (Prevnar 7, Pfizer, Inc., New York, NY) protects against 7 pneumococcal strains, which together account for 60.3% of invasive pneumococcal cases among children under 14 years of age in China [17].

China has successfully reduced the incidence of vaccine-preventable diseases through its government-subsidized Expanded Program on Immunization (EPI) [18]. The Chinese Advisory Committee on Immunization Practice recommends which vaccines should be included in the EPI and therefore offered for free to all Chinese children [19], but they do not issue formal guidelines on for-fee vaccines, such as Hib vaccine and PCV (Table 1). The relatively high market price of these for-fee vaccines is beyond the financial means of many Chinese [16], [20] and, consequently, members of the floating population and residents of rural areas have low coverage for vaccines they pay for themselves [21]–[23].

**Table 1:** Vaccines^a^ available in Shanghai, China, in 2012.

Vaccines in the Expanded Program on Immunization (EPI)	For-fee vaccines^b^
Hepatitis B vaccine	*Haemophilus influenzae* type b conjugate vaccine
Bacillus Calmette-Guérin	7-valent pneumococcal conjugate vaccine
Oral poliovirus vaccine	23-valent pneumococcal polysaccharide vaccine
Diphtheria-tetanus-pertussis vaccine	Rotavirus vaccine
Measles-rubella vaccine	Varicella vaccine
Measles-mumps-rubella vaccine	Influenza vaccine
Japanese encephalitis vaccine	
Group A meningococcal polysaccharide vaccine	
Group A+C meningococcal polysaccharide vaccine	
Hepatitis A vaccine	

a Vaccine list is non-exhaustive.

b In addition, many vaccines on the EPI list are also available for a fee from international vaccine manufacturers.

Shanghai is the largest and wealthiest city in China with 23 million residents, including 9 million members of the floating population, according to the 2010 China Census [24]. At immunization clinics throughout the 17 districts of Shanghai, vaccines from the EPI are offered free of charge to children in both the local and floating populations. For-fee vaccines are available at these same clinics. Since 2010, immunization clinics have uploaded records of the administration of both free and for-fee vaccines, along with children's demographic information, to the Shanghai Immunization Program Information System (SIPIS). All areas of Shanghai except Minhang district, Zhabei district, and parts of Pudong district use SIPIS. Vaccine records from 2005 to 2010, prior to implementation of the system, have been added to the database.

There is currently discussion within China about the advisability of funding Hib vaccine and PCV as part of the EPI [16]. Little information exists on population coverage of these vaccines in China, although such baseline measures are essential if the Chinese government decides to fund these vaccines or wishes to identify high risk groups for targeted interventions. The aim of this paper is to characterize population-level pediatric coverage of Hib vaccine and PCV in Shanghai in the year 2012, while also evaluating the interaction between residency and urbanicity on vaccine coverage.

## Materials and Methods

In this cohort study, a sample of children from SIPIS was selected by ordering the observations by birth year and parents' phone number. Parents' phone number was used in the selection process in order to list the children in a random fashion. All children in the database were eligible for inclusion in the study. The phone number was removed from the dataset before it was provided to the authors for analysis. The only potentially identifiable information in the analyzed dataset was date of birth. From an annual birth cohort of around 200,000 children, approximately 5,000 children were selected for each birth year from 2005 to 2010. Subsequently, 2,262 children (i.e., those born after August 1, 2010) were excluded because they were too young to be included in the study. The sample size of 5,000 children for each birth year was chosen to sufficiently power the analysis of a number of vaccination outcomes.

Selected children were entered into a dataset with the following SIPIS variables: sex, birthdate, resident district, residency (local or floating population), and vaccination dates. Both locals and the floating population were additionally categorized by urbanicity. Urbanicity, as defined by the Shanghai Centers for Disease Control and Prevention, was based on the child's resident district: urban districts included Changning, Huangpu, Hongkou, Jing'an, Putuo, Xuhui, and Yangpu, and suburban districts were Baoshan, Chongming, Fengxian, Jiading, Jinshan, Pudong, Qingpu, and Songjiang. Year of birth was dichotomized into a birth cohort variable (2005 through 2007 and 2008 through 2010) to reflect uncertainty in the rate of vaccine uptake over time and to more easily show important changes in vaccine coverage over a longer time period. The year 2008 was chosen as the cut point because PCV was introduced into China that year and because this point divides the study population approximately in half. One child without residency recorded was excluded.

Three measures of vaccination status were considered: dose 1 vaccination, series completion, and timeliness of dose 1 vaccination. Series completion was based on manufacturers' instructions and varied by child's age at dose 1 vaccination. For children whose Hib vaccine dose 1 was administered at <1 year, series completion comprised 2 primary doses and a booster after 1 year of age. For children whose first dose was administered at ≥1 year, only 1 dose was needed for series completion. For PCV, children whose dose 1 was administered at <7 months needed 3 primary doses and a booster after 1 year of age; children whose first dose was administered at 7 to <12 months needed 2 primary doses and a booster after 1 year; children whose first dose was administered at 1 to <2 years needed vaccination with dose 2 at least 2 months after dose 1; and children whose first dose was administered at ≥2 years required 1 dose. Timeliness of dose 1 vaccination was based on manufacturers' instructions: Hib vaccine administered at age 38 to 123 days was considered timely, and PCV administered at age 84 to 123 days was considered timely.

The diphtheria-tetanus-pertussis (DTP) vaccine, an EPI vaccine, was considered complete if the fourth dose was given at >12 months after completion of the 3-dose primary series.

Several observations in the dataset included vaccination dates in the 1980s, before children were born, and were re-coded as no vaccination. Analysis that included these observations did not yield substantially different point estimates.

Vaccination status was compared across demographic variables. The P-values in the bivariable analyses were calculated using Pearson's chi-square test of independence or Fisher's Exact Test. Binomial regression models accounted for the interaction between urbanicity and residency and produced the difference in vaccination coverage between locals and the floating population [25], but a model of PCV timely dose 1 was not constructed because of the low outcome count. The fit of different models was compared using a likelihood ratio test. All tests for significance were two sided and used an α level of 0.05. A plot of the cumulative incidence of dose 1 of Hib vaccine, PCV, and DTP was stratified across residency and urbanicity. All analyses used SAS version 9.2 (SAS Institute, Inc., Cary, North Carolina).

### Ethics statement

This study was deemed exempt from Institutional Review Board oversight at the University of Michigan and the Shanghai Centers for Disease Control and Prevention because it was limited to analysis of previously collected data.

## Results

Records for 28,141 children in SIPIS were included in the analysis. Of these children, 53.8% were male, 32.0% had urban residence, and 41.5% were locals (Table 2).

**Table 2:** Demographic characteristics, birth year, and residency status of the study population, Shanghai, China, 2012.

	Total population	Among locals	Among the floating population
	Count (percent)	Count (percent)	Count (percent)
**Overall**	28,141	11,691	16,450
**Sex**			
Male	15,141 (53.8%)	6, 007 (51.4%)	9,134 (55.5%)
Female	12,991 (46.2%)	5,678 (48.6%)	7,313 (44.5%)
Missing value	9		
**Birth year**			
2005	5,009 (17.8%)	2,129 (18.2%)	2,880 (17.5%)
2006	5,031 (17.9%)	2,168 (18.5%)	2,863 (17.4%)
2007	5,109 (18.2%)	2,289 (19.6%)	2,820 (17.1%)
2008	5,125 (18.2%)	2,074 (17.7%)	3,051 (18.6%)
2009	5,111 (18.2%)	1,965 (16.8%)	3,146 (19.1%)
2010	2,756 (9.8%)	1,066 (9.1%)	1,690 (10.3%)
**Urbanicity**			
Urban	9,013 (32.0%)	5,269 (45.1%)	3,744 (22.8%)
Suburban	19,128 (68.0%)	6,422 (54.9%)	12,706 (77.2%)

### Hib vaccine

**Figure 1:**
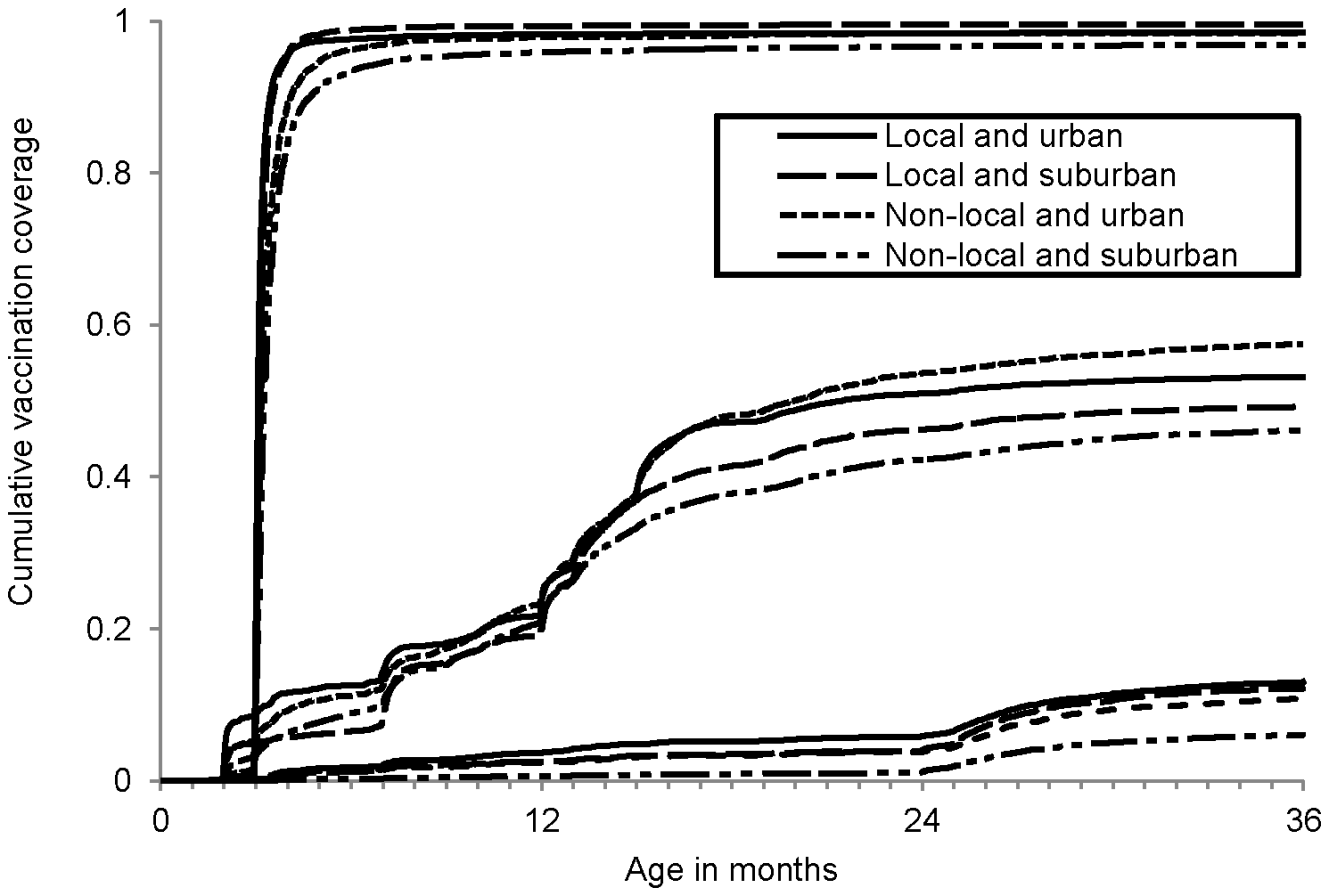
Cumulative vaccination coverage by vaccine. Age at administration of diphtheria-tetanus-pertussis vaccine (DTP) dose 1, Haemophilus influenzae type b (Hib) vaccine dose 1, and 7-valent pneumococcal conjugate vaccine (PCV) dose 1, stratified by residency and urbanicity, in Shanghai children, 2012.

Overall coverage of Hib vaccine dose 1 was 50.9%; urban dwellers (55.6%) had higher coverage than suburban dwellers (48.6%), and coverage was slightly higher in locals (51.8%) than in the floating population (50.7%) (Table 3). Allowing for interaction between residency and urbanicity, floating population children had 5.4% higher dose 1 vaccination coverage in urban districts (95% confidence interval (CI): 3.3%, 7.4%), and 3.0% lower coverage in suburban districts (95% CI: −4.5%, −1.5%). Figure 1 shows a plot of the cumulative vaccination coverage across age: floating population urban dwellers had the highest coverage, and floating population suburban dwellers had the lowest coverage.

**Table 3:** Coverage of dose 1, series completion, and timely dose 1 for Hib vaccine and PCV by sex, birth year and residency status of Shanghai children, 2012.

	Hib vaccine dose 1	Hib vaccine series completion^a^	Hib vaccine timely dose 1^b^	PCV dose 1	PCV series completion^a^	PCV timely dose 1^b^
	(n = 28,141)	(n = 14,316)	(n = 28,141)	(n = 28,141)	(n = 3,204)	(n = 28,141)
**Overall**	50.9%**	79.7%**	8.2%**	11.4%**	91.3%**	0.5%**
**Sex**						
Male	51.2%**	78.5%**	7.8%**	10.9%**	91.8%**	0.5%**
Female	50.5%**	81.0%**	7.3%**	12.0%**	90.9%**	0.5%**
**Birth cohort**						
2005–2007	48.9%**	83.3%**	4.4%**	10.4%**	97.3%**	0.0%**
2008–2010	53.2%**	75.8%**	11.3%**	12.5%**	82.5%**	1.1%**
**Urbanicity**						
Urban	55.6%**	81.6%**	10.7%**	14.3%**	88.6%**	0.8%**
Suburban	48.6%**	78.6%**	6.1%**	10.0%**	93.1%**	0.4%**
**Residency**						
Local	51.8%**	85.4%**	8.4%**	14.7%**	91.1%**	0.9%**
Floating	50.2%**	75.4%**	7.0%**	9.1%**	91.5%**	0.2%**

Hib: *Haemophilus influenzae* type b; PCV: 7-valent pneumococcal conjugate vaccine.

*P<0.05, chi-square test of homogeneity across demographic characteristic.

**P<0.0001, chi-square test of homogeneity across demographic characteristic or Fisher's Exact Test.

a Series completion was calculated for only those children with dose 1; variable constructed from manufacturers' recommendations.

b Timeliness was constructed from manufacturers' recommendations.

Most children vaccinated with Hib vaccine completed the series (79.7%). Series completion was higher in urban (81.6%) than suburban dwellers (78.6%) and in locals (85.4%) than in the floating population (75.4%) (Table 3). After adding in interaction between residency and urbanicity, locals and the floating population had lower series completion in both urban areas (−5.0%, 95% CI: −7.2%, −2.9%) and suburban areas (−11.2%, 95% CI: −12.8%, −9.6%) (Table 4).

**Table 4:** Differences between demographic groups in the coverage of dose 1, timely dose 1, and series completion for Hib vaccine and PCV in Shanghai children, 2012.

	Hib vaccine dose 1	Hib vaccine series completion^a^	Hib vaccine timely dose 1^b^	PCV dose 1	PCV series completion^a^
	(n = 28,141)	(n = 14,316)	(n = 28,141)	(n = 28,141)	(n = 3,204)
**Crude model**					
Floating vs. locals	−1.6%**	−10.0%**	−1.4%**	−5.6%**	0.4%**
**Adjusted model 1**					
Floating vs. locals	−0.1%**	−9.0%**	−0.8%**	−5.0%**	−0.6%**
Suburban vs. urban	−7.2%**	0.0%**	−3.7%**	−3.4%**	2.6%**
2008–2010 vs. 2005–2007	4.5%**	−6.1%**	6.6%**	−2.5%**	−14.6%**
**Adjusted model 2** c					
Floating vs. locals	5.4%**	−5.0%**	−2.3%**	−1.9%**	−2.7%**
Suburban vs. urban	−3.0%**	2.5%**	−4.6%**	−0.8%**	1.6%**
Floating * suburban	−8.4%**	−6.1%**	1.9%**	−4.6%**	2.6%**
2008–2010 vs. 2005–2007	4.7%**	−5.9%**	6.6%**	−2.4%**	−14.6%**

Hib: Haemophilus influenzae type b; PCV: 7-valent pneumococcal conjugate vaccine.

*P<0.05, chi-square test from binomial regression with identity link.

**P<0.0001, chi-square test from binomial regression with identity link.

a Series completion was calculated for only those children with dose 1; variable constructed from manufacturers' recommendations.

b Timeliness was constructed from manufacturers' recommendations.

c The likelihood ratio test comparing adjusted models 1 and 2 was not significant for the outcome “PCV series completion,” indicating that the fit did not improve with the addition of the interaction term.

Only 8.2% of children received a timely Hib vaccine dose 1. Timely administration was higher in urban dwellers (10.7%) than suburban dwellers (6.1%) and in locals (8.4%) than in the floating population (7.0%) (Table 3). Allowing for interaction between residency and urbanicity, floating population children had 2.3% lower timeliness in urban districts (95% CI: −3.5%, −1.0%) and similar timeliness in urban and in suburban districts (Table 4).

### PCV

Overall, 11.39% of children were vaccinated with PCV dose 1. Coverage in urban dwellers (14.3%) was higher than in suburban dwellers (10.0%), and locals (14.7%) had higher coverage than the floating population (9.1%) (Table 3). Adding in the interaction term between residency and urbanicity, the floating population had lower vaccination coverage in both urban areas (−1.9%, 95% CI: −3.3%, −0.4%) and suburban areas (−6.4%, 95% CI: −7.4%, −5.5%) (Table 4). Figure 1 shows the cumulative vaccination coverage of PCV dose 1; floating population suburban dwellers have noticeably lower vaccination coverage than the other demographic groups.

Of those with a first dose of PCV, 91.3% completed the series. Series completion was lower in urban (88.6%) than suburban dwellers (93.1%) and was similar between locals and the floating population (Table 3).

In the study population, 0.5% of the children had timely administration of PCV dose 1. Timeliness was higher in urban dwellers (0.8%) than suburban dwellers (0.4%). Locals (0.9%) were more likely to have timely administration of dose 1 vaccination than the floating population (0.2%) (Table 3).

### DTP

Coverage of DTP dose 1 was above 97% in all groups, regardless of residency or urbanicity status (Figure 1). Completion of the DTP series was greater than 98% in locals, 95.1% in floating population urban dwellers, and 89.9% in floating population suburban dwellers.

## Discussion

In response to the United Nation's fourth Millennium Development Goal of reducing mortality among children under 5 years of age, international organizations such as the Gates Foundation [26], the GAVI Alliance [27], and the WHO [2], [3], have funded the introduction of Hib vaccine and PCV in lower income countries. Despite the global emphasis on making these vaccines available, we found low coverage for PCV and Hib vaccine among children in Shanghai, a wealthy city in an upper middle income country.

The uptake of dose 1 for for-fee vaccines like PCV and Hib vaccine is a measure of Chinese citizens' willingness to pay for a vaccination. One study from 2011 in Tiantai county in Zhejiang province had Hib vaccine dose 1 coverage of 71.6%, compared to 50.9% in our study [28]; and in a national survey from 2011 of 4,681 children, dose 1 coverage was 45.3% for Hib vaccine and 9.9% for PCV, similar to the 11.4% coverage of PCV in our study [29]. Both studies corroborate our findings of low coverage for for-fee vaccines in China. Moreover, that Shanghai, the wealthiest city in China, does not have higher coverage for these vaccines than other regions indicates that financial factors are not the sole reason for low vaccination coverage and suggests that government intervention in vaccine marketing and financing will be needed to increase coverage. The uptake of government-funded vaccines in Shanghai is high: DTP dose 1 coverage was above 97% in our study and over 99% in Shanghai official records for 2011 [24]; our slightly lower estimate could be due to sampling error or the inclusion of more floating population records in SIPIS than in official records.

Vaccine series completion can serve as a useful proxy for the continuity of immunization services and is a key marker for adequate immunological protection [13], [30], although we were unable to locate any papers in the English or Chinese language literature which assessed Hib vaccine or PCV series completion in China. Zheng et al. stratified children by the number of doses they received and found that each immunized child had on average 2.04 doses of Hib vaccine and 1.16 doses of PCV [29]; compared to an evaluation of series completion, these findings have limited utility because the number of doses required for series completion depends on the age at which the child was given dose 1. Our study found high levels of series completion for both vaccines, indicating that once parents initiate the series, they are able to successfully overcome those barriers to vaccine receipt that contribute to low overall coverage. The high levels of completion also arise, in part, from children being vaccinated at an age when only one dose is required, which results in lower levels of vaccination timeliness.

Timeliness of dose 1 has been argued to be a more accurate estimator of the vaccinated proportion of the population [31] and a better indicator of health care utilization compared to dose 1 vaccination [32]. This is both because of the importance of providing immunological protection in infants as early as permissible and because past outbreaks have been tied to untimely vaccination [33], [34]. Unfortunately, very few children in our study had timely administration of Hib vaccine or PCV, though we did observe an increase in timely administration of Hib vaccine dose 1 from 4.4% in the 2005–2007 cohort to 11.3% in the 2008–2010 cohort. One study found higher timeliness of Hib vaccination in Germany than we did; in that study, on-time vaccination of Hib vaccine dose 1 increased from 12.5% in 1996 to 29.2% in 2003 [35].

The floating population generally had worse vaccination outcomes than locals. However, that there was high coverage and timeliness of DTP dose 1 across all demographic groups indicates that the floating population is clearly accessing services at immunization clinics. Understandably, they would be less likely to receive for-fee vaccines possibly because of cost and more limited availability. Few other studies in China have evaluated coverage of both EPI and for-fee vaccines across demographic groups. Chen found that coverage of for-fee vaccines was at least 10% lower in rural areas than urban areas, but no difference was observed between urban and rural areas in the coverage of EPI vaccines [23]. Thus, urbanicity in their study, like residency in ours, explained differences in coverage of for-fee vaccines but not in coverage of EPI vaccines.

Although we did not find important disparities between locals and the floating population in DTP dose 1coverage, other studies in China have suggested that the floating population has relatively low coverage of free vaccines compared to locals. For example, Wang et al. remarked that low coverage of measles vaccine, a free, EPI vaccine, in the floating population has resulted in that population serving as a reservoir for measles and, as a result, they should be targeted for increased immunization efforts [36]. In Korla City, Xinjiang Autonomous Region, Sun found that only two-thirds of floating population children had coverage of five EPI vaccines (Bacillus Calmette-Guérin, oral polio vaccine, DTP, measles vaccine, and hepatitis B vaccine), and that the government should accordingly provide more funding for vaccination outreach programs in the floating population [37].

PCV dose 1 coverage was much lower than Hib vaccine dose 1 coverage, likely because PCV has been more recently introduced in China and because the cost of PCV is much greater. That the floating population has comparatively worse timeliness and completion outcomes for PCV than the Hib vaccine would suggest that their access to recently introduced medical therapeutics is more limited.

This study has several limitations. Although there is not a standardized definition of floating population, some studies in the Chinese literature split residency into 3 categories: long-term residents, temporary residents, and the floating population [9], [38]; we were unable to make this distinction with the available data. Supplemental information on length of stay in Shanghai could improve the categorization of individuals into “floating population.” Additionally, more individual-level data, such as parents' education or profession, could permit better characterization of this population. Han et al., for example, found that among floating population children, those whose father had a college education had higher coverage of EPI vaccines (95.5%) than those whose fathers only had a high school (92.6%) or elementary (80.0%) education [22]. Sun et al. similarly found that, among the floating population, children whose primary caregiver had a high school education had 3.24 times higher odds of having timely coverage of EPI vaccines than children whose caregivers were less educated [21]. The relative heterogeneity of the floating population in our study could help partially explain why they had higher Hib vaccine dose 1 coverage than locals in urban areas.

Only data from single antigen Hib vaccines were used in this study. Since 2010, combination vaccines with Hib have been introduced into Shanghai, and consequently our coverage results for Hib vaccination may be an underestimate.

This paper was limited to data from immunization clinics and therefore may be subject to coverage bias. In their study of the floating population in Beijing, Sun et al. found that 11.8% of children in this population did not access immunization clinics, and their vaccination outcomes were worse than children who did access the clinics [21]. Thus, our results may overestimate vaccination coverage, particularly among the floating population.

Finally, our data were derived from Shanghai, a particularly wealthy urban area in China, and findings may not be generalizable to more rural areas or to western regions. Other large, urban conglomerations on the eastern coast of China, such as Beijing, Tianjin, and Guangzhou, which all have significant communities of floating populations, are more appropriate comparisons to Shanghai. Nonetheless, as previously mentioned, our PCV and Hib vaccine dose 1 coverage estimates are similar to a national survey in China [29].

In conclusion, this study found low coverage and poor timeliness of Hib vaccine and PCV among young children in Shanghai. As a result, a large cohort of infants remains at risk of developing invasive Hib and pneumococcal disease which continue to be important causes of childhood morbidity and mortality. Socioeconomically marginalized groups in Shanghai, such as the floating population and suburban dwellers, may have higher Hib and pneumococcal disease risk compared to locals and urban dwellers. Future studies of Hib and pneumococcal invasive disease in Shanghai should assess whether the disparity in vaccination coverage reported here translates into significantly different rates of serious illness in these subpopulations. Vaccine cost and awareness are probably the greatest barriers to uptake in suburban dwellers and among the floating population. Future studies could qualitatively explore parental attitudes towards vaccination and evaluate non-governmental interventions to increase vaccine uptake. In addition, these studies should collect more detailed demographic information on individuals to further determine what specific groups should be targeted for interventions and what factors are associated with vaccination. Placing Hib vaccine and PCV on the EPI list, which would allow for government funding support, would be the most effective way to increase uptake. However, it will continue to be important to ensure that timeliness of vaccination and completeness of the vaccination series is equitable across demographic groups.
